# Antiplatelet Therapy in Patients With Abdominal Aortic Aneurysm Without Symptomatic Atherosclerotic Disease

**DOI:** 10.1001/jamanetworkopen.2023.39715

**Published:** 2023-10-25

**Authors:** Chalotte W. Nicolajsen, Mette Søgaard, Martin Jensen, Nikolaj Eldrup, Torben B. Larsen, Samuel Z. Goldhaber, Christian-Alexander Behrendt, Peter B. Nielsen

**Affiliations:** 1 Department of Cardiology, Aalborg University Hospital, Aalborg, Denmark; 2 Aalborg Thrombosis Research Unit, Department of Clinical Medicine, Faculty of Health, Aalborg University, Aalborg, Denmark; 3 Department of Vascular Surgery, Viborg Regional Hospital, Viborg, Denmark; 4 Department of Vascular Surgery, Rigshospitalet, Copenhagen, Denmark; 5 Institute of Clinical Medicine, Copenhagen University, Copenhagen, Denmark; 6 Thrombosis Research Group, Division of Cardiovascular Medicine, Brigham and Women’s Hospital, Boston, Massachusetts; 7 Harvard Medical School, Boston, Massachusetts; 8 Department of Vascular and Endovascular Surgery, Asklepios Medical School, Asklepios Clinic Wandsbek, Hamburg, Germany; 9 Division of Cardiovascular Medicine, Brandenburg Medical School Theodor Fontane, Neuruppin, Germany

## Abstract

**Importance:**

Patients with abdominal aortic aneurysm have a high risk of ischemic events associated with concomitant atherosclerotic cardiovascular disease, and current clinical practice guidelines recommend antiplatelet therapy to mitigate this risk. However, in patients with aneurysms without symptomatic atherosclerosis, the benefit of antiplatelet therapy has been sparsely investigated.

**Objective:**

To estimate the effect of antiplatelets on the risk of ischemic events and bleeding in individuals with abdominal aneurysms with no symptomatic atherosclerotic vascular disease.

**Design, Setting, and Participants:**

A comparative effectiveness research study using a target trial emulation framework was performed. Population-based, cross-linked observational data from Danish national health registries containing comprehensive, individual-level information on all Danish citizens were used to evaluate patients who were antiplatelet-naive and diagnosed with abdominal aortic aneurysms, with no record of symptomatic atherosclerotic vascular disease, from January 1, 2010, through August 21, 2021.

**Exposure:**

Prescription filled for aspirin or clopidogrel.

**Main Outcomes and Measures:**

Risk of ischemic events (myocardial infarction and/or ischemic stroke) and risk of major bleeding. For target trial emulation, trials were emulated as sequential, contingent on patient eligibility at the time of inclusion, and were evaluated by means of pooled logistic regression models to estimate the intention-to-treat and as-treated effects, expressed as hazard ratio (HR) and event-free survival.

**Results:**

A total of 6344 patients (65.2% men; age, 72 [IQR, 64-78] years) provided 131 047 trial cases; 3363 of these cases involved initiation of antiplatelet therapy and 127 684 did not. A total of 182 ischemic events occurred among initiators and 5602 ischemic events occurred among noninitiators, corresponding to an intention-to-treat HR of 0.91 (95% CI, 0.73-1.17) and an estimated absolute event-free survival difference of −0.6% (95% CI, −1.7% to 0.5%). After censoring nonadherent person-time, the treatment HR was 0.90 (95% CI, 0.68-1.20), with similar risk difference. For bleeding, the intention-to-treat HR was 1.26 (95% CI, 0.97-1.58) and the event-free survival difference was 1.0%. The treatment HR was 1.21 (95% CI, 0.82-1.72); the risk difference was similar.

**Conclusions and Relevance:**

In this study, no evidence of effectiveness of antiplatelet therapy to lower the risk of ischemic events and a trend toward higher bleeding risk was noted. The observed differences between the treatment groups were minimal, suggesting limited clinical relevance of antiplatelet treatment.

## Key Points

**Question:**

Do patients with abdominal aortic aneurysms without symptomatic atherosclerosis benefit from antiplatelet therapy?

**Findings:**

In this comparative effectiveness research study using target trial emulation methods including 6344 patients with 131 047 trial cases, limited effectiveness of antiplatelets to lower the risk of ischemic events was noted. The absolute risk differences between the treatment groups were minimal.

**Meaning:**

The results of this study suggest limited clinical relevance of treatment and warrant caution in prescribing prophylactic antiplatelet therapy for patients with abdominal aortic aneurysms without symptomatic atherosclerosis.

## Teaser

This comparative effectiveness study examines the use of antiplatelet therapy in a target trial emulation of patients with abdominal aortic aneurysm who had no symptoms of atherosclerotic vascular disease.

## Introduction

Abdominal aortic aneurysm (AAA) has been associated with a 2-fold increase in risk of cardiovascular ischemic events compared with individuals without AAA.^1^ Coexisting cardiovascular disease is one of the key determinants of prognosis during surveillance and post-AAA repair.^2,3^ Observational studies suggested higher survival among patients with AAA receiving antiplatelet therapy.^4,5^ Accordingly, clinical guidelines recommend antiplatelet therapy for patients presenting with AAA, particularly in the presence of symptomatic atherosclerotic disease.^6,7^ However, the proportion of patients with AAA with a record of symptomatic atherosclerosis is decreasing,^8^ and the benefits and risks associated with antiplatelet prophylaxis in those without symptomatic atherosclerotic disease are still not clear.

To our knowledge, there have been no randomized clinical trials (RCTs) assessing the efficacy and safety of antiplatelet therapy vs placebo in patients with AAA, and current recommendations are accordingly based on level B/C evidence.^6^ At the same time, pharmacologic prophylaxis with antiplatelets in patients with AAA who do not exhibit symptoms of atherosclerosis is controversial, considering the associated risk of bleeding and a potential limited clinical benefit of antiplatelet therapy in individuals with asymptomatic atherosclerosis.^7,9,10,11^

To bridge this knowledge gap, we designed a target trial using a target trial emulation framework^12^ with the purpose of estimating the effect of antiplatelet therapy vs no antiplatelet therapy on the risk of ischemic events (myocardial infarction [MI] and ischemic stroke) and bleeding in a cohort of patients with AAA without concomitant symptomatic atherosclerotic vascular disease. We emulated the trial using observational health care data with complete national coverage.^13,14^

## Methods

We designed and specified a target trial and emulated the trial, using observational data from Danish national health registries, which are linked using the unique identity number assigned to all residents at birth or immigration. The registries contain comprehensive longitudinal, individual-level information on all Danish citizens, enabling data collection with almost no missing data or loss to follow-up.^15^ The analysis and results were reported in accordance with The Reporting of Studies Conducted Using Observational Routinely-Collected Health Data (RECORD) Statement. This study was conducted in accordance with the General Data Protection Regulation and the North Denmark Region’s record of processing activities. Data were provided by the Danish Health Data Authority. According to Danish law, ethical approval is not required for studies based on pseudonymized health register data.

### Target Trial and Target Trial Emulation

We developed a protocol for a hypothetical target trial that could have answered the causal question of interest to investigate the average estimated treatment effect of antiplatelet therapy vs no treatment on the incidence of ischemic events and bleeding in patients who were antiplatelet-naive with AAA disease without a diagnosis of symptomatic atherosclerotic cardiovascular diseases. Specifications of trial components are listed in the eMethods and eTable 1 in Supplement 1. Briefly, trial eligibility criteria included a diagnosis of AAA, age between 50 and 90 years, no previous antiplatelet treatment, no other indication for antiplatelet therapy (any history of atherosclerosis, peripheral arterial occlusive disease, ischemic heart disease, ischemic stroke, or transient ischemic attack), and no contraindications for antiplatelet therapy. Eligible patients were followed up for a maximum of 5 years.

We used data from the Danish national health registries for target trial emulation and mirrored the target trial components, with modifications required to accommodate the use of observational data. We retrieved information on diagnoses, hospitalizations, and outpatient visits from the Danish National Patient Registry (DNPR). The DNPR holds information on hospitalizations since 1977 and outpatient visits at all hospitals in Denmark since 1995. Data include Civil Personal Registry numbers, dates of admission and discharge, primary and up to 20 secondary diagnoses coded by the *International Statistical Classification of Diseases and Related Health Problems, Tenth Revision* (*ICD-10*).^16^ Diagnoses used for definition of eligibility have been previously validated with high positive predictive values (>90%).^17^ Data on prescriptions fills were retrieved from the Danish National Prescription Registry, which holds detailed information on the purchase date, Anatomical Therapeutic Chemical classification code, package size, and dose for every prescription fill since 1994.^18^ Data on patient characteristics, such as age, sex, and vital status, were extracted from the Civil Registration System.^19^

### Study Population and Exposure

**Figure 1.**
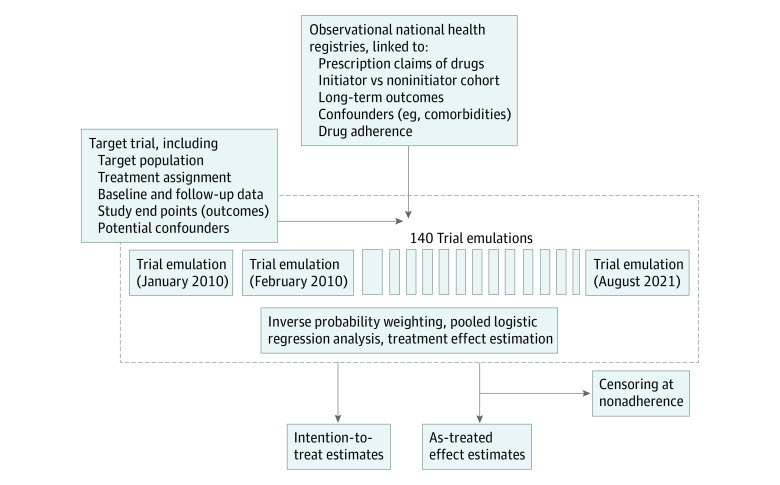
Study Design and Data Processing in Relation to Target Trial Specifications and Trial Emulation

We applied trial eligibility criteria to individuals registered in the DNPR between January 1, 2010, and August 21, 2021. Individuals could potentially meet the eligibility criteria multiple times during the study period. To enable inclusion of patients eligible more than once, and to include noninitiators who later began antiplatelet therapy, we emulated the target trial as a sequence of trials, with a new trial starting each month, similar to previous study designs in other disease areas.^20^ This allowed individuals to contribute information to multiple trials, depending on eligibility status.^12,13^ Based on the number of months from January 1, 2010, through August 21, 2021 (end of data), 140 trials were constructed. Screening for inclusion was assessed in the first (baseline) month of each of the sequential trials, and eligible patients were assigned to a treatment group and followed up in the registries. Individuals with an outcome in the first trial month were excluded. An illustration of target trial emulation and data flow are provided in Figure 1, while details on the flowchart of inclusions are provided in eFigure 1 and specifications on *ICD-10* and Anatomical Therapeutic Chemical codes in eTable 2 in Supplement 1.

Among patients with AAA who were antiplatelet-naive, we compared 2 treatment strategies of initiation of antiplatelet therapy (aspirin, 75-150 mg, or clopidogrel, 75 mg) or no initiation of antiplatelet therapy. Individuals were classified as initiators or noninitiators according to the treatment strategy, compatible with the data at baseline (ie, prescription fill of antiplatelet therapy within the baseline trial month). To emulate randomization, exchangeability between groups was assumed conditional on the propensity for receiving the observed treatment.

### Outcome Measures

The primary outcome was risk of ischemic events, defined as a composite outcome of a diagnosis of myocardial infarction (MI) and/or ischemic stroke. Secondary outcomes were risk of MI and ischemic stroke separately and a safety outcome of major bleeding (defined as bleeding events leading to hospital contact) (eTable 2 in Supplement 1). All outcome diagnoses have been validated in the DNPR with positive predictive values greater than 90% for all ischemic and bleeding outcomes.^17,21^ Follow-up started at the end of the baseline month of every sequential new trial and ended at the first outcome event, death, loss to follow-up, 5 years after baseline, or administrative end of data.

#### Estimation of the Observational Analogue to ITT

In the target trial emulation, the indicator for treatment was defined as initiation of antiplatelet therapy within the baseline month. Confounding control was handled by means of propensity scores that were derived to calculate stabilized inverse probability of treatment weights (IPTW).^22^ Calculation of the propensity score included age (continuous and squared terms), sex, relevant comorbidity (hypertension, diabetes, atrial fibrillation, kidney disease, and heart failure), medical treatment with other cardioprotective agents (statins and antihypertensive therapy), and a proxy for smoking status (diagnosis of smoking-related chronic obstructive pulmonary disease, tobacco abuse/registered smoking status, smoking cessation advice, or prescription fill of medicine for smoking cessation). eFigure 1 in Supplement 1 provides a causal directed acyclic graph depicting the underlying potential confounders of the exposure-outcome association. Because of the incremental covariate time related to the multiple trial eligibilities for the same individual, we decided post hoc to include time since first AAA diagnosis (restricted cubic spline) in the final model.

We estimated the average treatment effect as the difference in 5-year risk of outcomes, expressed as hazard ratios (HRs) and depicted by event-free survival curves.^23^ Time was modeled separately and included in the pooled logistic regression in the estimation of HR as a product term between exposure group and time to model the individual baseline hazard.^23^ We used nonparametric bootstrapping with 500 samples to calculate the SE and 95% CIs for the main analyses.

#### Estimation of the Observational Analogue to As-Treated

The as-treated analyses investigated the observed effect under continuous adherence to the treatment assigned in the baseline trial month. Briefly, individuals in the treatment arm were censored if they discontinued antiplatelet therapy, whereas individuals in the nontreatment arm were censored if they initiated antiplatelet therapy. Discontinuation was defined as the end of the data for the last available tablet, based on a daily dose calculation with a maximum gap between daily doses of 60 days (grace period). The as-treated analytic approach was performed with adjustments for the same baseline covariates as applied in the ITT analyses, and postbaseline (time-varying) covariates, including hypertension, diabetes, atrial fibrillation, kidney disease, heart failure, medical treatment with other cardioprotective agents (statins and antihypertensive therapy), and smoking.

#### Subgroup and Sensitivity Analyses

A priori, we selected clinically relevant subgroups for whom estimated treatment effect could potentially be different and performed analyses stratified by (1) with or without concomitant statin therapy, (2) restricting the analyses to include only aspirin as exposure (as opposed to the main analysis including aspirin or clopidogrel), (3) patients aged 80 years or older, and (4) excluding patients with a cancer diagnosis within the past 5 years. We also conducted sensitivity analyses by prolonging the discontinuation gap between daily doses of prescription fills to 90 days only for the as-treated analyses. We then conducted analyses in a cohort restricted to patients with a maximum of 6 months between AAA diagnosis and study inclusion. For all subgroup and sensitivity outcomes, we used robust variance estimates when calculating the 95% CIs, which may yield more conservative estimates. Significance testing was not conducted.

### Statistical Analysis

Baseline demographic characteristics and comorbidities are presented descriptively for the trial populations. Summarized categorical data are reported as percentages and continuous data as medians (IQRs). Outcomes under exposure were contrasted by pooling the emulated trials and by fitting a pooled logistic regression model of each effect estimate (intention-to-treat [ITT] and treatment). Analyses were performed using SAS, version 9.4 (SAS Institute Inc), R, version 4.2.2 (R Foundation for Statistical Computing), and Stata/MP, version 17 (StataCorp LLC).

## Results

A total of 25 326 individuals were registered with a diagnosis of AAA between January 1, 2010, and August 21, 2021. Of these, 6344 individuals (25.0%; 65.2% men; 34.8% women; median age, 72 [IQR, 64-78] years) met eligibility criteria at least once during the study period. As individuals could be eligible for multiple sequential trials, the patients contributed a total of 131 047 individual trial cases, of which 3363 initiated antiplatelet therapy and 127 684 did not receive antiplatelet therapy (eTable 3 in Supplement 1). Figure 1 shows the data flow according to study design, while the flowchart in eFigure 2 in Supplement 1 summarizes inclusions and exclusions of the trial population.

Among initiators of antiplatelet therapy, 3166 participants (94.1%) initiated aspirin, while 197 individuals (5.9%) initiated clopidogrel. In the IPTW population, the median age was 72.0 (IQR, 64.0-78.0) years, 34.7% were women, 19.0% were registered smokers, 7.4% had a history of diabetes, 34.6% received concomitant statin therapy, and 51.1% received concomitant antihypertensive therapy (Table 1). After the IPTW was applied, the standardized mean differences in baseline characteristics were below 0.1 (eFigure 3 in Supplement 1). eTable 4 in Supplement 1 summarizes baseline characteristics for the unweighted cohort.

**Table 1.** Baseline Characteristics of the Trial Population After IPTW

Characteristic	No. (%)			Standardized difference^a^
	Total (N = 131 047)	Noninitiators (n = 127 684)	Initiators (n = 3363)	
Sex				
Female	45 403 (34.6)	44 215 (34.6)	1188 (35.3)	0.003
Male	85 644 (65.4)	83 469 (65.4)	2175 (64.7)	
Age, median (IQR), y	72.0 (64.0-78.0)	72.0 (64.0-78.0)	72.0 (66.0-77.0)	0.072
Time since AAA diagnosis, mean (SD), d	1117 (1054)	1137 (1054)	356 (771)	0.846
Smoking^b^	24 350 (18.6)	23 701 (18.6)	648 (19.3)	0.010
Comorbidity				
Hypertension	31 590 (24.1)	30 739 (24.1)	851 (25.3)	0.019
Diabetes	5847 (4.5)	9385 (7.3)	252 (7.5)	<0.001
COPD	20 273 (15.5)	19 760 (15.5)	513 (15.3)	0.013
Chronic kidney disease^c^	891 (0.7)	867 (0.7)	24 (0.7)	0.002
Heart failure	1934 (1.5)	1882 (1.5)	51 (1.5)	0.002
Atrial fibrillation	2315 (1.8)	2250 (1.8)	65 (1.9)	0.011
Venous thromboembolism	4381 (3.3)	4287 (3.4)	94 (2.8)	0.036
Major bleeding^d^	7040 (5.4)	6896 (5.4)	144 (4.3)	0.056
Obesity^e^	4863 (3.7)	4741 (3.7)	122 (3.6)	0.007
Medical treatment				
Aspirin (in baseline month)	3166 (94.1)	NA	3166 (94.1)	NA
Clopidogrel (in baseline month)	197 (5.9)	NA	197 (5.9)	NA
Statin^f^	44 901 (34.2)	43 720 (34.2)	1181 (35.1)	0.007
Antihypertensive^f^	65 176 (49.7)	63 428 (49.7)	1748 (52.0)	0.030
Antidiabetic^f^	8422 (6.4)	8209 (6.4)	213 (6.3)	0.008

Abbreviations: AAA, abdominal aortic aneurysm; IPTW, inverse probability of treatment weights; NA, not applicable.

^a^ Values less than 0.1 indicate no significant difference between groups.

^b^ Registered smokers, previous and current.

^c^ Disease registered more than 5 years prior to inclusion.

^d^ Gastrointestinal, intracranial, and other major bleeding registered more than 6 months prior to inclusion.

^e^ Registered body mass index greater than 25 (calculated as weight in kilograms divided by height in meters squared).

^f^ Prescription fill within 1 year prior to inclusion.

### Effect Estimates for Ischemia

**Figure 2.**
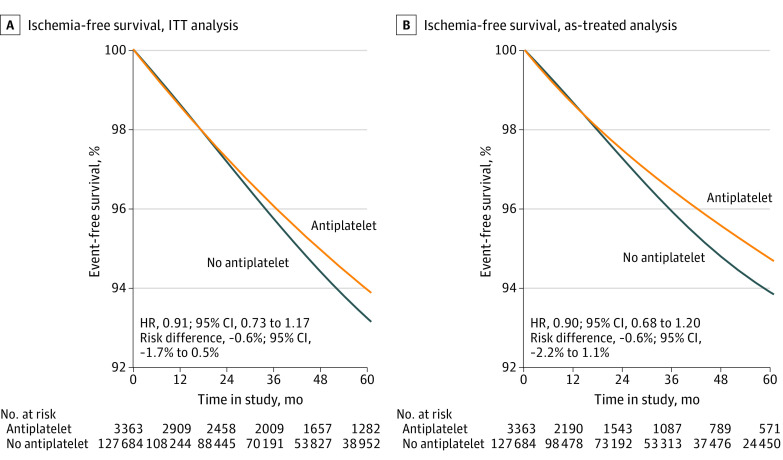
Ischemia-Free Survival Estimates for Intention-to-Treat (ITT) and Treatment Analyses HR indicates hazard ratio; 95% CIs were determined using nonparametric bootstrapping with 500 samples.

During a total of 5 011 227 person-months of follow-up, 182 ischemic events were observed among antiplatelet initiators, while 5602 ischemic events were observed among those who did not initiate antiplatelet therapy (further information on the original population in eTable 3 in Supplement 1). Median follow-up duration was 24 (IQR, 11-40) months for initiators and 23 months (IQR, 11-38) months for noninitiators. Table 2 displays the risks of ischemic events between antiplatelet therapy initiators and noninitiators at 5 years’ follow-up. In the ITT analyses, the HR for ischemic events was 0.91 (95% CI, 0.73-1.17), with an event-free survival difference of −0.6% (95% CI, −1.7% to 0.5%) between the 2 groups (Figure 2). For the separate outcomes of MI and ischemic stroke, the ITT HR was 0.81 (95% CI, 0.57-1.23) for MI and 0.95 (95% CI, 0.73-1.17) for stroke, and the event-free survival difference was −0.7% (95% CI, −1.6% to 0.6%) for MI and −0.3% (95% CI, −1.2% to 0.7%) for stroke (Table 2). Following censoring for nonadherent trial participation, 114 ischemic events were observed among antiplatelet initiators and 4331 ischemic events were observed among noninitiators during a median follow-up of 18 (IQR, 8-34) months for initiators and 20 (IQR, 9-35) months for noninitiators. The as-treated HR was 0.90 (95% CI, 0.68-1.20) (Table 2), and the 5-year survival difference was −0.6% (95% CI, −2.2% to 1.1%) (Figure 1).

**Table 2.** Estimated Intention-to-Treat and As-Treated HRs of Ischemic Events and Major Bleeding Outcomes With vs Without Antiplatelet Therapy

Outcome	Target trial emulation^a^									
	Estimated intention-to-treat effect					Estimated as-treated effect				
	Initiator (n = 3363)		Noninitiator (n = 127 684)		Effect estimate, HR (95% CI)	Initiator (n = 3363)		Noninitiator (n = 127 684)		Effect estimate, HR (95% CI)
	No. of events	Event-free survival	No. of events	Event-free survival		No. of events	Event-free survival	No. of events	Event-free survival	
Ischemic events	182	0.937	5602	0.931	0.91 (0.73-1.17)	114	0.943	4331	0.937	0.90 (0.68-1.20)
Myocardial infarction	66	0.977	2264	0.972	0.81 (0.57-1.23)	40	0.979	1904	0.972	0.76 (0.50-1.24)
Ischemic stroke	118	0.959	3442	0.957	0.95 (0.73-1.17)	74	0.965	2465	0.964	0.96 (0.73-1.20)
Major bleeding	129	0.947	3212	0.958	1.26 (0.97-1.58)	86	0.951	2349	0.961	1.21 (0.82-1.72)

Abbreviation: HR, hazard ratio.

^a^ Confidence intervals determined using nonparametric bootstrapping with 500 samples.

### Safety Estimates for Bleeding Events

**Figure 3.**
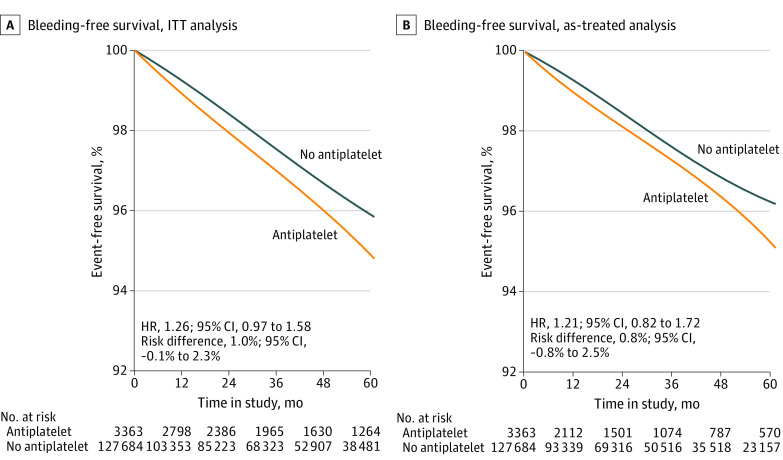
Bleeding-Free Survival Estimates for Intention-to-Treat (ITT) and Treatment Analyses HR indicates hazard ratio. Point estimates were determined using regression; 95% CIs were determined using nonparametric bootstrapping with 500 samples.

For the safety outcome of bleeding, 129 events occurred among initiators and 3212 events among noninitiators during follow-up (eTable 3 in Supplement 1). The ITT HR for major bleeding events was 1.26 (95% CI, 0.97-1.58) (Table 2). The event free survival difference between initiators and noninitiators was 1.0% (95% CI, −0.1% to 2.3%) at 5 years’ follow-up (Figure 3). The as-treated analytic approach, with censoring of nonadherent person-time, yielded similar effect estimates (as-treated HR, 1.21; 95% CI, 0.82-1.72), with an event-free survival difference of 0.8% (95% CI, −0.8% to 2.5%) (Table 2).

### Subgroup and Sensitivity Analyses

Subgroup analyses, especially analyses restricted to comparison of aspirin vs no aspirin therapy, showed results generally comparable to those found in the main analyses with minimal risk differences (eTable 5 in Supplement 1). For patients receiving concomitant statin therapy, we observed no significant difference, with an HR of 1.02 (95% CI, 0.77-1.35) for ischemic events and an HR of 1.11 (95% CI, 0.78-1.58) for bleeding events, whereas for those not using statin therapy, the difference was more pronounced, yet risk differences were less than 1%. For patients aged 80 years or older, data on only 568 initiators and 31 655 noninitiators were available for analyses and showed a higher risk of ischemic events (ITT HR, 1.11; 95% CI, 0.75-1.63) and a higher risk of bleeding (ITT HR, 1.12; 95% CI, 0.69-1.82); however, the difference in event-free survival was marginal.

Sensitivity analyses revealed similar results with marginal differences between groups (eTable 6 in Supplement 1). Restricted to patients with a maximum of 6 months between AAA diagnosis and inclusion, the ITT HR was 0.84 (95% CI, 0.69-1.01) for ischemia and 1.10 (95% CI, 0.88-1.38) for major bleeding.

## Discussion

In this target trial emulation including patients with AAA without symptomatic atherosclerosis, we observed a trend toward lower risk of ischemic events among those receiving antiplatelet therapy but a higher risk of major bleeding. However, the risk difference was minimal (−0.6% for ischemic events and 1.0% for major bleeding over 5 years’ follow-up), indicating minor clinically relevant differences.

To our knowledge, this is the largest-scale study to formally investigate the estimated effectiveness of antiplatelet therapy vs no treatment on the risk of ischemic events and bleeding in an AAA population without symptomatic atherosclerosis. Our findings are consistent with the outcomes from RCTs investigating the efficacy of aspirin in the general population without atherosclerotic manifestations.^10,11^ The ARRIVE trial, a multinational RCT including 12 546 patients with an estimated moderate to high 10-year risk (10%-20%) of ischemic events, found an HR of 0.96 (95% CI, 0.81-1.13) for ischemic events and 2.11 (95% CI, 1.36-3.28) for major bleeding.^10^ The ASCEND trial, including 15 480 participants with diabetes and no cardiovascular atherosclerosis, found an HR of 0.98 (95% CI, 0.80-1.19) for MI, 0.88 (95% CI, 0.73-1.06) for ischemic stroke, and 1.29 (95% CI, 1.09-1.52) for major bleeding.^11^

Based on results from the ARRIVE and ASCEND trials, suggesting no or moderate ischemic risk reduction at the expense of an increased risk of bleeding, recommendations have changed for patients without manifest atherosclerosis.^10,11,24,25^ Antiplatelet therapy is no longer recommended routinely but is considered beneficial only in specific patients at very high cardiovascular risk and with minimal risk of bleeding.^26,27,28^ These revisions provoke critical inquiries, raising the question of whether in light of the current AAA guidelines,^6,7^ the concept of very high cardiovascular ischemic risk for all patients with AAA should be taken into account for the possible advantage of antiplatelet therapy. Alternatively, the question arises as to whether it is more prudent to heed the contemporary guidelines on primary cardiovascular disease prevention for patients without atherosclerotic manifestations and restrict the recommendation of antiplatelet therapy to patients with AAA who have symptomatic atherosclerotic cardiovascular disease.^26,28^ The present study adds to the understanding of benefit and risk associated with use of antiplatelet therapy and allows us to estimate differences in the risk of outcomes with treatment vs no treatment.

The current recommendations on antiplatelet therapy in AAA management are based on evidence extrapolated from trials investigating secondary prevention in atherosclerotic cardiovascular disease and from retrospective studies. They suggest lower all-cause mortality among patients with AAA receiving antiplatelet therapy.^6,7^ Yet, a more recent meta-analysis found no survival difference associated with antiplatelet therapy in patients with small AAAs (defined by an aneurysm diameter between 3 and 5.5 cm), with an HR of 0.91 (95% CI, 0.75-1.11), while antiplatelet therapy was associated with lower all-cause mortality in a patients undergoing AAA repair (HR, 0.84; 95% CI, 0.76-0.92).^29^ Data available for the meta-analyses were, however, based on low-quality observational studies with high risk of bias.^29^ Given the current limited evidence for a beneficial effect of antiplatelet therapy, our findings, based on nationwide high-quality data, add to the evidence against recommending antiplatelet therapy for patients with AAA without symptomatic atherosclerosis. A dedicated trial investigating the effect of antiplatelet therapy in patients with AAA with and without symptomatic atherosclerosis is still warranted. Yet, based on the number of outcome events registered in our study, sample size calculations indicate the need of including more than 5000 individuals in such a trial (2500 in each treatment arm) to identify a difference in the risk of ischemic events. We envision that such a trial is not likely to be conducted and highlights the importance of providing profound evidence from other sources.

### Strengths and Limitations

The main strengths of this study were the large sample size, the broad variety of available variables, the long follow-up period, and the high validity of inclusion and outcome diagnoses retrieved from the DNPR.^17,30^ The completeness of data available from the Danish health registries allowed us to quantify the association between antiplatelet therapy and clinical outcomes, with adjustments for a wide range of confounding factors. Furthermore, it allowed for analyses of rare, and potentially late, outcomes, such as major bleeding and effect measures based on adherence to treatment. Our study design of emulating a hypothetical trial is a useful framework for comparative effectiveness research using observational data to mitigate confounding, immortal time bias, and selection bias between exposure groups.^30^

The design, however, also comes with certain limitations. A key challenge in analyses using data from routine clinical practice is that treatment is not randomly assigned, possibly resulting in biased effect estimates from unmeasured confounding factors. In clinical practice, physicians may tend to initiate treatment in patients with expected high ischemic risk, but at the same time with a low risk of bleeding. Therefore, the as-treated estimates particularly may be subject to selection bias, while a study without selection bias might find higher risk of bleeding. Successful emulation of randomization depends on adjustments for baseline confounders and the untestable assumption of no residual confounding. Our estimates are limited to covariates available from the Danish health registries. Differences in unmeasured characteristics, potentially confounding our results, cannot be excluded. In addition, since few patients initiated clopidogrel in this study, conclusions on the effectiveness of this drug specifically are limited.

## Conclusions

In this comparative effectiveness study using target trial emulation methods of the estimated effect of antiplatelet therapy in patients with AAA without concomitant symptomatic atherosclerotic vascular disease, the absolute differences in risk between initiators and noninitiators of antiplatelet therapy were negligible, indicating no clinically meaningful difference. Overall, these findings do not support recommendations of prophylactic antiplatelet therapy in this target population. Along with the absence of high-level evidence on antithrombotic strategies, this study highlights the necessity for an RCT. In the meantime, careful consideration in prescribing prophylactic antiplatelet therapy should be given for all patients with AAA.

## Notes

Supplement 1. **eMethods.** Specifications of Target Trial Protocol **eTable 1.** Protocol Components of Target Trial and Trial Emulation **eTable 2.** Definition of Variables **eTable 3.** Number of Individuals, Trial Cases and Outcome Events Included in Analyses **eTable 4.** Baseline Characteristics of the Study Population Before IPTW **eTable 5.** Intention-to-Treat Estimates of Outcomes (Ischemic and Bleeding Events) in Subgroups **eTable 6.** Sensitivity Analyses With Estimates of Outcomes (Ischemic and Bleeding Events) **eFigure 1.** Directed Acyclic Graph of Potential Confounding Factors **eFigure 2.** Flowchart of Inclusion **eFigure 3.** Plot of Maximum Standardized Difference of Baseline Covariates of Study-Population Before and After Applying IPTW

Supplement 2.
Data Sharing Statement
