# Supplementary material for: Antiplatelet Therapy in Patients With Abdominal Aortic Aneurysm Without Symptomatic Atherosclerotic Disease
Source: JAMA Netw Open. 2023 Oct 25;6(10):e2339715. doi: 10.1001/jamanetworkopen.2023.39715 (PMC10600585; doi:10.1001/jamanetworkopen.2023.39715)
Supplement: Supplement 1. — eMethods. Specifications of Target Trial Protocol eTable 1. Protocol Components of Target Trial and Trial Emulation eTable 2. Definition of Variables eTable 3. Number of Individuals, Trial Cases and Outcome Events Included in Analyses eTable 4. Baseline Characteristics of the Study Population Before IPTW eTable 5. Intention-to-Treat Estimates of Outcomes (Ischemic and Bleeding Events) in Subgroups eTable 6. Sensitivity Analyses With Estimates of Outcomes (Ischemic and Bleeding Events) eFigure 1. Directed Acyclic Graph of Potential Confounding Factors eFigure 2. Flowchart of Inclusion eFigure 3. Plot of Maximum Standardized Difference of Baseline Covariates of Study-Population Before and After Applying IPTW [file jamanetwopen-e2339715-s001.pdf]

Supplementary Online Content

Nicolajsen CW, Søgaard M, Jensen M, et al. Antiplatelet therapy in patients with abdominal aortic aneurysm without symptomatic atherosclerotic disease. *JAMA Netw Open*. 2023;6(10):e2339715. doi:10.1001/jamanetworkopen.2023.39715

eMethods. Specifications of Target Trial Protocol

eTable 1. Protocol Components of Target Trial and Trial Emulation

eTable 2. Definition of Variables

eTable 3. Number of Individuals, Trial Cases and Outcome Events Included in Analyses

eTable 4. Baseline Characteristics of the Study Population Before IPTW

eTable 5. Intention-to-Treat Estimates of Outcomes (Ischemic and Bleeding Events) in Subgroups

eTable 6. Sensitivity Analyses With Estimates of Outcomes (Ischemic and Bleeding Events)

eFigure 1. Directed Acyclic Graph of Potential Confounding Factors

eFigure 2. Flowchart of Inclusion

eFigure 3. Plot of Maximum Standardized Difference of Baseline Covariates of Study-Population Before and After Applying IPTW

This supplementary material has been provided by the authors to give readers additional information about their work.

© 2023 Nicolajsen CW et al. *JAMA Network Open.*

eMethods. Specifications of target trial protocol

A target trial was specified with the following components (resumé in eTable 1)

Eligibility criteria. Enrollment of patients with a diagnosis of AAA, aged ≥50 and ≤ 90 years between January 2010 and August 2021, who had no manifestations of symptomatic peripheral arterial disease (PAD); intermittent claudication, rest pain, ischemic ulcers and gangrene, ischemic heart disease (IHD); angina, previous myocardial infarction (MI), manifestations of chronic myocardial ischemia, or ischemic stroke. Participants must not have any contraindications for antiplatelet therapy (major liver- or kidney impairment within the last 5 years of inclusion, recent major bleeding, or prevalent anticoagulant (OAC) treatment (within 6 months of inclusion), and no history of cancer (except non-melanoma skin-cancer) within 5 years from inclusion. Participants must not have used antiplatelet therapy (including ticagrelor and prasugrel) within the last 12 months prior to inclusion.

Baseline was defined as the first month of randomization when all eligibility criteria were met.

Treatment strategies. 1. Usual medical care or 2. Initiation of antiplatelet therapy (aspirin or clopidogrel, type of antiplatelet at the discretion of the physician). When clinically warranted during the follow-up, patients and their physicians will decide whether to start, stop or switch therapy.

Treatment assignment. Each eligible participant will be randomly assigned to a strategy with initiation of treatment within 1 month from randomization. Patients with events of interest within this period will be excluded. Patients will be aware of the strategy to which they are assigned.

Outcomes. Outcomes of interest is a composite of MI and ischemic stroke. Secondary outcome is MI and ischemic stroke as separate outcomes as well as major bleeding, defined as any bleeding requiring hospital contact.

Follow-up. All participants will be followed from baseline and until first ischemic stroke, MI, death, loss to follow-up, or administrative end of follow-up (five years or august 2021), whichever happens first.

Causal contrasts. The intention-to-treat (ITT) effect of being assigned to antiplatelet therapy versus no initiation of antiplatelet therapy at baseline and the On-treatment (OT) effect of antiplatelet initiation and continuation over follow-up.

Statistical analysis. In both ITT and OT analyses, we will use pooled logistic regression analysis to estimate the effect of antiplatelet therapy via comparison of 5-year risk of outcome expressed as hazard ratios and standardized survival curves.[1] For ITT analyses we fitted a pooled logistic regression with an indicator for of assigned strategy and a flexible function of months since randomization. For OT analyses we fitted a model after censoring participants if and when they deviate from assigned treatment. Time-varying stabilized inverse-probability weights will be used to adjust for time-varying confounding associated with adherence and outcome of interest.

To identify potential subgroups for whom treatment may potentially be more beneficial, stratified analyses will be conducted based on baseline information on age (>= 80 years), with and without concomitant statin therapy and according to type of antiplatelet (aspirin/ clopidogrel).

© 2023 Nicolajsen CW et al. *JAMA Network Open.*

eTable 1. Protocol Components of target trial and trial emulation

Component	Target trial specification	Target trial emulation
**Eligibility criteria**	Diagnosis of AAA, age ≥ 50 years and ≤ 90 years, no previous symptomatic PAD, IHD, CVD, no use of antiplatelet therapy [a], no contraindications for antiplatelet therapy [b], no prevalent cancer [c]<br><br>Baseline defined as day of randomization.	Same as target trial<br><br>Baseline defined as the first of every trial month where all eligibility criteria were met, yielding potentially 140 sequential trial months for each participant.
**Treatment strategies**	i) Initiation of antiplatelet therapy (aspirin, clopidogrel)<br><br>ii) No initiation of antiplatelet<br><br>Within 30 days from randomization	Same as target trial.<br>Study participants were classified according to treatment strategy at baseline, assuming exchangeability.<br>Initiation of treatment defined as +/- prescription claim of antiplatelet (aspirin or clopidogrel) within 30 days from baseline (first trial month)
**Treatment assignment**	Non-blinded random assignment	Assignment according to treatment vs. no treatment in first trial month<br><br>Randomization was emulated by adjusting for baseline confounders
**Outcomes**	1. Composite of MI and ischemic stroke<br>2. Major bleeding	Same as target trial
**Follow-up**	Starts at randomization, ends at first outcome diagnosis, death, loss to follow-up or administrative censoring (emigration, end of study)<br><br>Patients were followed for up to five years	Same as target trial
**Causal contrasts**	Intention-to-treat (ITT) effect<br>On-treatment (OT) effect	Observational analogue
**Statistical analyses**	ITT + OT analyses	Pooled logistic regression analyses<br>ITT: Adjustment for baseline prognostic factors associated with the probability of initiating antiplatelet therapy to emulate randomization<br><br>OT: Censuring at deviation from assigned treatment<br>Adjustments for baseline and time-varying prognostic factors associated with treatment adherence

AAA; abdominal aortic aneurysmal disease, PAD; peripheral arterial disease (intermittent claudication, ischemic rest pain, ischemic ulceration, or gangrene), IHD; ischemic heart disease (angina, myocardial infarction), CVD; cerebrovascular disease (ischemic stroke or transient ischemic attack), OAC; oral anticoagulation therapy, MI; myocardial infarction, DD; daily dose. [a]Use of aspirin, clopidogrel, ticagrelor, or prasugrel within 12 months prior to randomization, [b] Contraindications (major liver- and/or kidney deficiency or haemodialysis < five years before randomization, major bleeding < six months before and/or OAC therapy < 12 months prior to randomization), [c] All cancer (except non-melanoma skin cancer) diagnosed within five years before randomization.

© 2023 Nicolajsen CW et al. *JAMA Network Open.*

eTable 2. Definition of variables

Diagnosis / treatment	ICD	ATC
**Inclusion**		
Abdominal aortic aneurysm	I713, I714	
**Exclusion**		
Ischemic heart disease	I20, I21	
Angina	I20	
Myocardial infarction	I21	
Ischemic cerebrovascular disease		
Ischemic stroke	I63	
Transient ischemic attack	G45	
Peripheral atherosclerotic arterial disease	I702, I739A, I739C	
Claudication	I739A	
Restpain, gangrene	I739C, I702	
Major liver impairment	B150, B160, B162, B190, K704, K72, K766, I85	B150 B160 B162 B190 K704 K72 K766 I85
Major renal impairment (stage 5)	N185	
Major acute renal impairment	N17	
Major bleeding		
Intracranial	I60, I61, I62	
Gastrointestinal	I850I 864A K226 K228F K250 K252 K254 K256 K260 K262 K264 K266 K270 K272 K274 K276 K280 K282 K284 K286 K290 K298A K625 K638B K638C K661 K838F K868G K920 K921 K922	
Other (minus nosebleed and traumatic bleeding)	E078B E274B G951A I230 I312 I319A J942 M250	
**Outcome**		
Ischemic stroke	I63	
Myocardial infarction	I21	
**Other comorbidity**		
Diabetes	E10-14, H360, O240-43	A10A, A10B<br>≥ 1 category
Hypertension	I10-13, I15	
Chronic obstructive pulmonary disease	J40-J47, J60-J67, J684 J701 J703 J841 J920 J921 J982 J983	
Chronic obstructive pulmonary disease, smoking-related	J42, J44, J684, J841	
Tobacco abuse	F17, T652, Z716, Z720A, Z720E	
**Treatment (drugs)**		
Antiplatelet		B01AC04, B01AC06
Aspirin		B01AC06
Clopidogrel		B01AC04

© 2023 Nicolajsen CW et al. *JAMA Network Open.*

**Diagnosis / treatment**	**ICD**	**ATC**
Oral anticoagulant		
Coumarin		B01AA01-04
Direct oral anticoagulant		B01AE07, B01AF01, B01AF02, B01AX05
Other		
Statin		C10
Antihypertensive		C02, C03D, C07, C08, C09
Smoking cessation		N06AX12, N07BA01, N07BA03,
**Procedures**		
Hemodialysis		BJFD2
Smoking cessation interviews		BQFT01, BQFS01 ZZP01A1A, ZZP0020

© 2023 Nicolajsen CW et al. *JAMA Network Open.*

eTable 3. Number of individuals, trial cases and outcome events included in analyses

		Initiators of antiplatelets	Non-initiators of antiplatelets
**Original population**			
Unique individuals		3363	4987
Unique events	Ischemic events	182	354
	Bleeding events	129	238
**Weighted pseudo-population**			
Trials cases		3363	127684
Trial events	Ischemic events	182	5602
	Bleeding events	129	3212

© 2023 Nicolajsen CW et al. *JAMA Network Open.*

eTable 4. Baseline characteristics of the study population before IPTW

	Non-initiators % (N)	Initiators % (N)	Std Diff
N	127,684	3,363	-
Sex, female	34.9 (44599)	23.2 (780)	0.26
Age, Median (IQR)	72.0 (64.0-78.0)	72.0 (66.0-77.0)	0.05
Time since AAA diagnosis, days	1138 (1054)	341 (760)	0.87
Registered smokers, previous/current	18.6 (23797)	15.7 (528)	0.08
**Comorbidity**			
Hypertension	24.2 (30867)	20.2 (681)	0.10
Diabetes mellitus	7.4 (5720)	6.8 229)	0.02
COPD	15.5 (19837)	12.5 (420)	0.09
COPD, smoking-related	12.4 (15871)	10.8 (362)	0.05
Chronic renal disease[a]	0.7 (867)	0.7 (23)	< 0.01
Heart failure	1.5 (1880)	1.5 (52)	0.01
Atrial fibrillation	1.8 (2256)	1.6 (53)	0.02
Venous thromboembolism	3.4 (4288)	2.6 (88)	0.04
Major Bleeding[b]	5.4 (6904)	4.0 (134)	0.07
Obesity[c]	3.7 (4761)	2.9 (96)	0.05
**Medical treatment**			
Statins [d]	34.2 (43684)	34.2 (1150)	< 0.01
Antihypertensives [d]	49.7 (63405)	50.2 (1687)	0.01
Antidiabetics [d]	6.4 (8222)	5.9 (198)	0.02

[a] Disease registered > 5 years prior to inclusion, [b]Gastrointestinal, intracranial and other major bleeding registered > 6 months prior to inclusion, [c] Registered BMI>25, [d]Prescription claim within 1 year prior to inclusion. Abbreviations: AAA; abdominal aortic aneurysm, Std Diff; standardized difference, IPTW; Inverse probability of treatment weights

© 2023 Nicolajsen CW et al. *JAMA Network Open.*

eTable 5. Intention-to-treat estimates of outcomes (ischemic and bleeding events) in subgroups

Subgroup	Treatment group	N	Ischemic events			Bleeding		
			Event-free survival	ITT HR (CI 95%)	Event-free survival difference, %	Event-free survival	ITT HR (CI 95%)	Event-free survival difference, %
**Aspirin-only**	Initiators	3652	0.936	0.87 (0.73-1.03)	-1.0	0.944	1.20 (0.97-1.49)	0.9
	Non-initiators	151231	0.927			0.953		
**Statin**	Initiators	1137	0.935	1.02 (0.77-1.35)	0.1	0.943	1.11 (0.78-1.58)	0.5
	Non-initiators	51672	0.936			0.948		
**No Statin**	Initiators	2548	0.929	0.90 (0.73-1.11)	-0.8	0.947	1.21 (0.69-1.56)	0.8
	Non-initiators	99326	0.922			0.956		
**Age ≥ 80 years**	Initiators	568	0.887	1.11 (0.75-1.63)	1.0	0.913	1.12 (0.69-1.82)	0.8
	Non-initiators	31655	0.897			0.922		
**No cancer last 5 years**	Initiators	3363	0.937	0.91 (0.76-1.09)	-0.6	0.947	1.26 (0.99-1.60)	1.0
	Non-initiators	127684	0.931			0.958		

CI- Confidence Interval (robust variance estimates)

© 2023 Nicolajsen CW et al. *JAMA Network Open.*

eTable 6. Sensitivity analyses with estimates of outcomes (ischemic and bleeding events)

Analysis	Treatment group	N	Ischemic events			Bleeding		
			Event-free survival	Hazard ratio (CI 95%)	Survival difference (%)	Event-free survival	Hazard ratio (CI 95%)	Survival difference (%)
**90 days gap in DD [a]**	Initiators	3363	0.937	0.94 (0.74-1.19)	-0.4	0.949	1.22 (0.92-1.62)	0.9
	Non-initiators	127684	0.934			0.958		
**AAA <= 6 months from inclusion[b]**	Initiators	2443	0.928	0.84 (0.69-1.01)	-1.4	0.947	1.10 (0.88-1.38)	0.5
	Non-initiators	20624	0.915			0.952		

CI- Confidence Interval, DD-Daily Dose, [a] Intention-to-treat analyses, [b] On-Treatment analyses

© 2023 Nicolajsen CW et al. *JAMA Network Open.*

eFigure 1. Directed Acyclic Graph of potential confounding factors

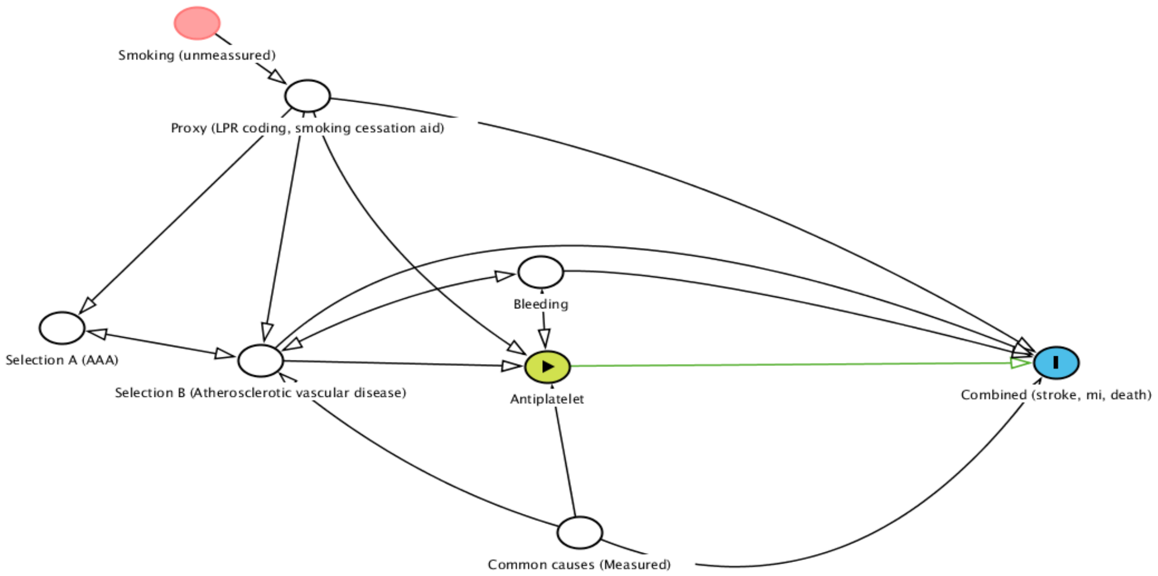

Common causes with association to exposure and outcome: sex, age, calendar year of inclusion, time since abdominal aortic aneurysm diagnosis, relevant comorbidity (diabetes, hypertension, atrial fibrillation, heart failure) use of other cardioprotective medication (statin, antihypertensives)

© 2023 Nicolajsen CW et al. *JAMA Network Open.*

eFigure 2. Flowchart of inclusion

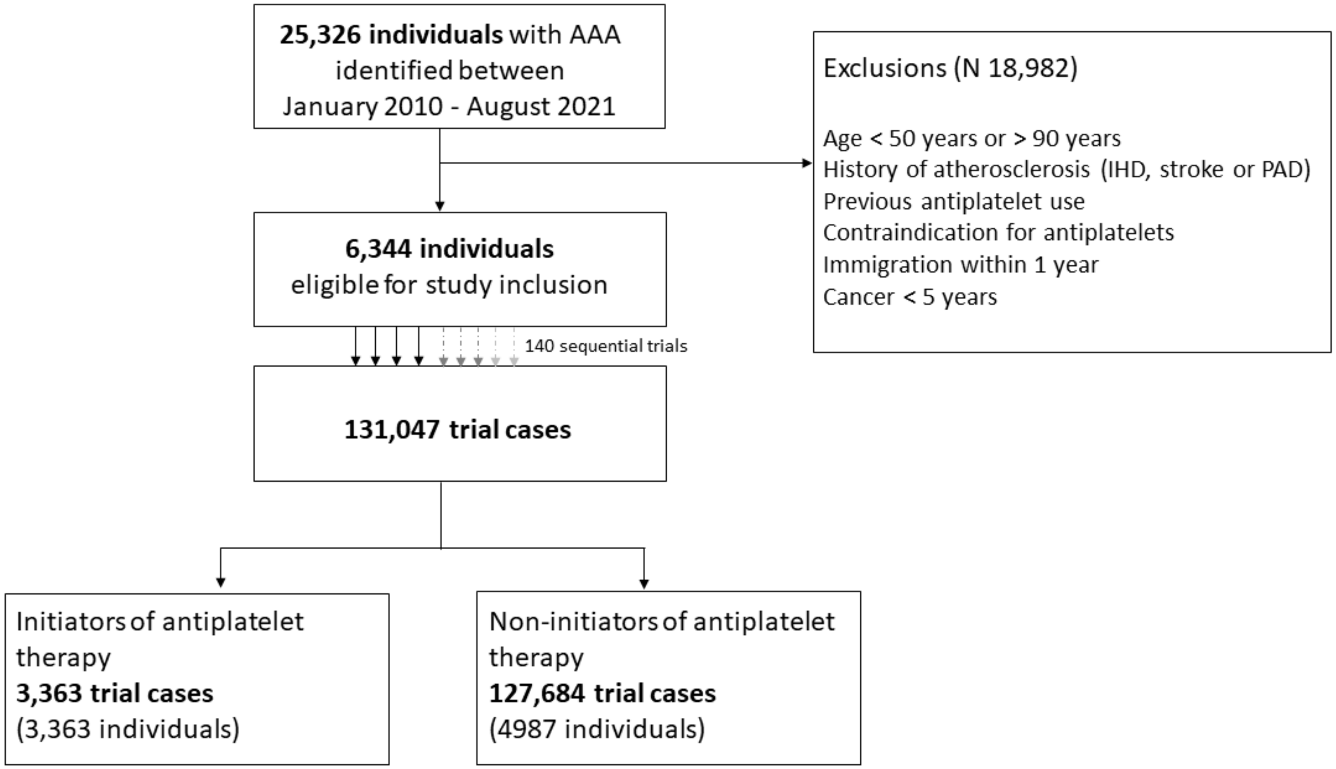

© 2023 Nicolajsen CW et al. *JAMA Network Open.*

eFigure 3. Plot of maximum standardized difference of baseline covariates of study-population before and after applying IPTW.

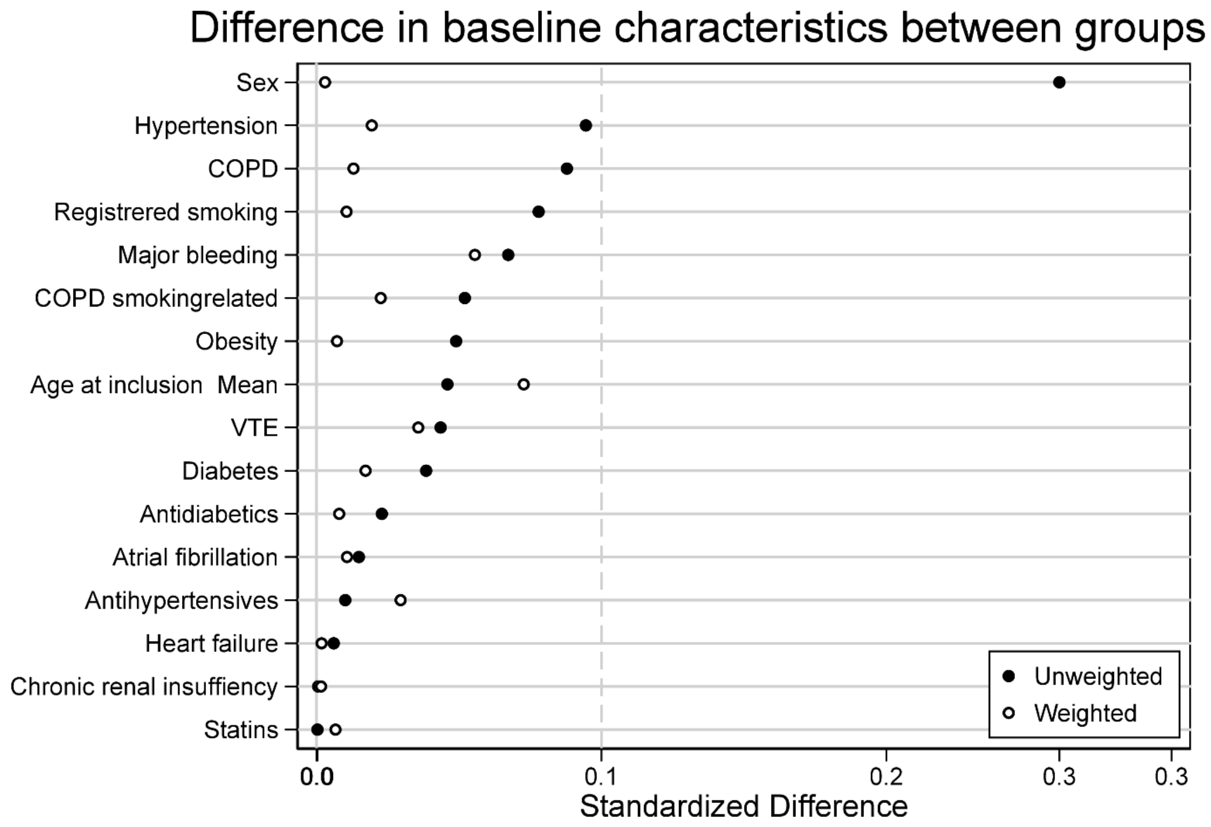

Abbreviations: IPTW - inverse probability weights of propensity scores, COPD – chronic obstructive pulmonary disease, VTE – venous thromboembolism

© 2023 Nicolajsen CW et al. *JAMA Network Open.*
