# Supplementary material for: Antiplatelet Therapy in Patients With Abdominal Aortic Aneurysm Without Symptomatic Atherosclerotic Disease
Source: JAMA Netw Open. 2023 Oct 25;6(10):e2339715. doi: 10.1001/jamanetworkopen.2023.39715 (PMC10600585; doi:10.1001/jamanetworkopen.2023.39715)
Supplement: Supplement 2. — Data Sharing Statement [file jamanetwopen-e2339715-s002.pdf]

Data Sharing Statement

Nicolajsen. Antiplatelet Therapy in Patients With Abdominal Aortic Aneurysm Without Symptomatic Atherosclerotic Disease. *JAMA Netw Open*. Published October 25, 2023. doi:10.1001/jamanetworkopen.2023.39715

Data
Data available: No

Additional Information
Explanation for why data not available: Because of the non-anonymized nature of the data collected for this study, requests to access the dataset from third parties are not allowed according to Danish data safety regulations.
